# Complete Analysis of the Epidemiological Scenario around a SARS-CoV-2 Reinfection: Previous Infection Events and Subsequent Transmission

**DOI:** 10.1128/mSphere.00596-21

**Published:** 2021-09-08

**Authors:** Laura Pérez Lago, Leire Pérez Latorre, Marta Herranz, Francisco Tejerina, Pedro J. Sola-Campoy, Jon Sicilia, Julia Suárez-González, Cristina Andrés-Zayas, Alvaro Chiner-Oms, Santiago Jiménez-Serrano, Neris García-González, Iñaki Comas, Fernando González-Candelas, Carolina Martínez-Laperche, Pilar Catalán, Patricia Muñoz, Darío García de Viedma

**Affiliations:** a Servicio de Microbiología Clínica y Enfermedades Infecciosas, Gregorio Marañón General University Hospital, Madrid, Spain; b Instituto de Investigación Sanitaria Gregorio Marañón (IiSGM), Madrid, Spain; c CIBER Enfermedades Respiratorias (CIBERES), Madrid, Spain; d Genomics Unit, Gregorio Marañón General University Hospital, Madrid, Spain; e Instituto de Biomedicina de Valencia-CSIC, Valencia, Spain; f Joint Research Unit Infection and Public Health FISABIO-University of Valencia, Institute for Integrative Systems Biology (I2SysBio), Valencia, Spain; g CIBER Salud Pública (CIBERESP), Madrid, Spain; h Servicio de Oncohematología, Gregorio Marañón General University Hospital, Madrid, Spain; i Departamento de Medicina, Universidad Complutense, Madrid, Spain; U.S. Centers for Disease Control and Prevention

**Keywords:** COVID-19, SARS-CoV-2, reinfection, transmission, genomics

## Abstract

The first descriptions of reinfection by SARS-CoV-2 have been recently reported. However, these studies focus exclusively on the reinfected case, without considering the epidemiological context of the event. Our objectives were to perform a complete analysis of the sequential infections and community transmission events around a SARS-CoV-2 reinfection, including the infection events preceding it, the exposure, and subsequent transmissions. Our analysis was supported by host genetics, viral whole-genome sequencing, phylogenomic viral population analysis, and refined epidemiological data obtained from interviews with the involved subjects. The reinfection involved a 53-year-old woman with asthma (Case A), with a first COVID-19 episode in April 2020 and a much more severe second episode 4-1/2 months later, with SARS-CoV-2 seroconversion in August, that required hospital admission. An extended genomic analysis allowed us to demonstrate that the strain involved in Case A’s reinfection was circulating in the epidemiological context of Case A and was also transmitted subsequently from Case A to her family context. The reinfection was also supported by a phylogenetic analysis, including 348 strains from Madrid, which revealed that the strain involved in the reinfection was circulating by the time Case A suffered the second episode, August-September 2020, but absent at the time range corresponding to Case A’s first episode.

**IMPORTANCE** We present the first complete analysis of the epidemiological scenario around a reinfection by SARS-CoV-2, more severe than the first episode, including three cases preceding the reinfection, the reinfected case *per se*, and the subsequent transmission to another seven cases.

## INTRODUCTION

On 24 August 2020, the first SARS-CoV-2 reinfection case (1) was reported in a resident of Hong Kong who traveled to Europe and was exposed to a strain/lineage different from the one identified in the first episode. Few SARS-CoV-2 reinfections have been published (2–9) since. With the available data, it is not possible to establish a common reinfection pattern between the affected subjects regarding age, time between episodes, and severity of the second episode with respect to the first.

Prolonged SARS-CoV-2 RNA shedding may extend up to 101 days (10). Whole-genome sequencing (WGS) allows distinction between extended shedding cases and reinfection by determining the genetic differences between the first and the second strains. Such differences may be significant, e.g., in cases affected by strains from different lineages (1), or moderate, yet enough to demonstrate that each strain followed a different evolutionary path (2). Moreover, the European Centre for Disease Prevention and Control has suggested the use of WGS to document reinfections, in an alternative way, by showing that the strain involved in the reinfection is clustered with other strains circulating in the context of exposure (11).

To date, reports on reinfection cases focus on the analysis of the case *per se*. Here, we describe a SARS-CoV-2 reinfection case in a subject without clinical risk factors and analyze the epidemiological scenario before and after the reinfection. We identify the exposure event responsible for the reinfection and describe the extensive transmission from the reinfected case to her relatives.

## RESULTS

A 53-year-old woman (Case A) was admitted to the emergency room in our hospital (Gregorio Marañón, Madrid, Spain) on 3 April 2020 presenting with dyspnea, fever, cough with expectoration (24 h), and a history of bronchial asthma. She had been treated regularly with inhaled budesonide, with no systemic corticosteroids between April and August 2020. At admission, HIV serology and IgG, IgM, and IgA determinations were requested with negative and normal results, respectively. Blood tests and chest X ray showed no outstanding changes. Positive SARS-CoV-2 PCR was obtained for the nasopharyngeal exudate (threshold cycle [*C_T_*] value 30). Since the patient had no respiratory failure or other serious conditions, she was discharged from hospital the same day and given symptomatic treatment. The patient remained symptomatic for over 2 weeks (mainly dyspnea and fever) with eventual resolution of symptoms. On 18 April, a second reverse transcriptase PCR (RT-PCR) was performed with a positive result. One month after discharge, the SARS-CoV-2 RT-PCR was repeated (12 May), and this time the result was negative. SARS-CoV-2 serology was not available at that time.

The source of infection was undetermined. Case A reported having been confined to her home with her husband during the 3 weeks prior to the beginning of symptoms and denied contact with anyone else during confinement. Her husband did not have any symptoms, and therefore, SARS-CoV-2 testing was not performed.

On 14 August, 4-1/2 months (140 days) from her first positive RT-PCR, Case A began to have fever, dyspnea, cough, and arthromyalgia. Another SARS-CoV-2 RT-PCR was performed (21 August) with a positive result (*C_T_* value 22).

On 25 August, Case A returned to the emergency department, this time with respiratory failure and multiple bilateral pulmonary infiltrates and was thus admitted to the hospital. No SARS-CoV-2 antibodies were detected in the admission tests. Upon admission, the presence of lymphopenia, mild hypertransaminasemia, and elevated lactate dehydrogenase (LDH) and C-reactive protein (CRP) stood out.

During the first 48 h, Case A showed radiological worsening and respiratory failure, experiencing significant bronchospasm. She was given corticosteroids, remdesivir, and lopinavir/ritonavir. Three RT-PCRs were performed during hospitalization (28 August and 2 and 6 September, all positive; *C_T_* values 21, 33, and 33, respectively). The patient progressively improved and was discharged 17 days after admission with oxygen therapy. Prior to discharge, the SARS-CoV-2 serology (SARS-CoV-2 IgG Architect; Abbott, Chicago, IL, USA) was repeated (8/9). The result was positive with titers of 7.04.

### Epidemiological events preceding reinfection.

**FIG 1:**
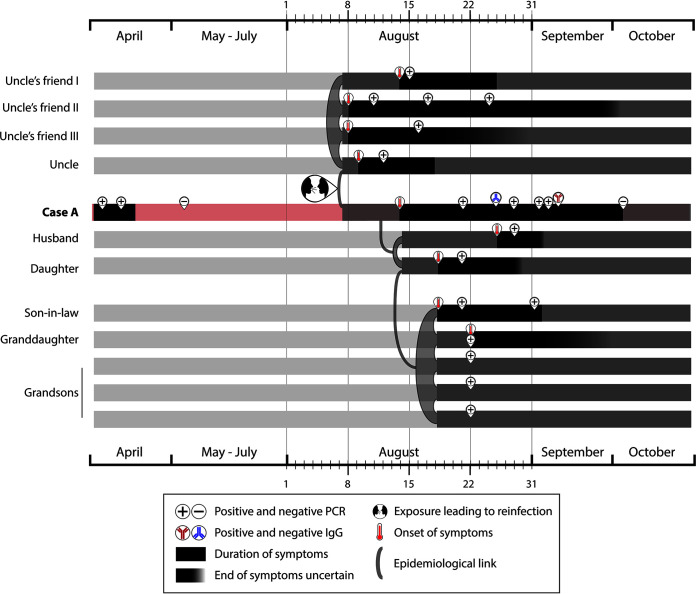
Time lapses between study cases indicating duration of symptoms, positive and negative RT-PCRs (when available), positive and negative serology (for Case A’s second episode), and contacts and exposures between cases.

Twelve days before (7 August) the onset of case A’s second episode, she had had close physical contact with an uncle (no face masks, tight physical contact that included kisses and hugs [Fig. 1]). Besides Case A, there was no close contact with the uncle by any of the other family members. Two days later (9 August), the uncle developed a cough, arthromyalgias, asthenia, and dysthermia. The RT-PCR SARS-CoV-2 test performed on 12 August was positive (*C_T_* value 19). His symptoms resolved on 18 August.

Case A was interviewed for more details regarding her and her uncle’s epidemiological context. Three of her uncle’s friends had also fallen ill with COVID-19; all four attended the same religious center every Friday and had had additional contacts. When interviewed, one of them said that the last time all four met was at the religious center on 7 August. The three friends had subsequent positive RT-PCRs (11, 15, and 16 August [Fig. 1]). Case A and her husband and daughter had not attended that religious center or met her uncle’s friends.

### Epidemiological events following reinfection.

On 25 August, 11 days after the onset of Case A’s second episode symptoms, her husband complained of fever and malaise. He tested positive for SARS-CoV-2 by PCR (*C_T_* value 25). On 28 August, his dyspnea worsened and an interstitial infiltrate was observed in the upper left lobe. Home isolation was indicated for 14 days.

On 18 August, Case A’s daughter, who visited her daily, started with a cough, odynophagia, asthenia, and fever of 39°C. On 21 August, she tested positive for COVID-19 (RT-PCR). Home isolation ended after 14 days, without RT-PCR control. The daughter’s husband began having symptoms the same day (18 August) and tested positive on 21 August (RT-PCR) and 2 September. The daughter’s husband had contact only with his wife and not with case A (Fig. 1). The RT-PCR tests performed on their four children were all positive, but only one of the children developed symptoms (starting on 22 August) (Fig. 1).

### Genomic analysis.

We first confirmed that the specimens isolated from Case A, who had had positive SARS-CoV-2 RT-PCR results in April (first episode) and August (second episode) belonged to the same patient, as indicated by the identical microsatellite short tandem repeat (STR)-PCR patterns obtained from the human DNA in the corresponding samples (see Fig. S1 in the supplemental material).

10.1128/mSphere.00596-21.1 FIG S1 Microsatellite STR-PCR analysis on Case A’s specimens confirming that the samples have a common origin. Download FIG S1, PDF file, 0.3 MB. Copyright © 2021 Pérez Lago et al. 2021 Pérez Lago et al. https://creativecommons.org/licenses/by/4.0/ This content is distributed under the terms of the Creative Commons Attribution 4.0 International license.

**FIG 2:**
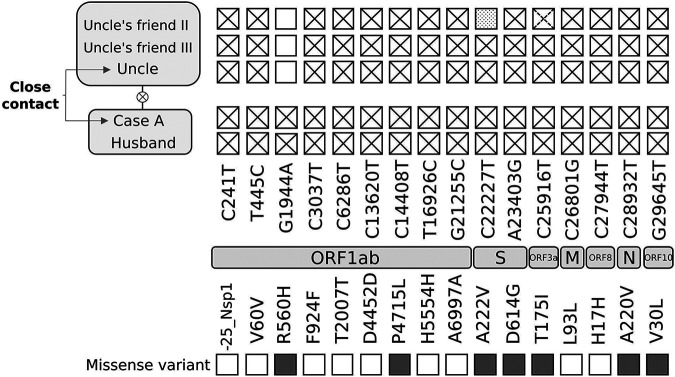
Single-nucleotide polymorphisms identified by whole-genome sequencing analysis for Case A and related cases. Genes where the single-nucleotide polymorphisms map and their nature and potential impact are indicated. The dashed cross indicates a heterozygous call at that position. The dotted square indicates an uncovered position.

Next, we compared the SARS-CoV-2 sequences of the strains isolated from Case A in the first and second episodes. The analysis of the specimen collected during episode 1 allowed us to confirm that the sequences corresponded to SARS-CoV-2, but it did not provide enough coverage (only 18% of the chromosome offered at least 30× coverage) to determine the complete consensus sequence and single-nucleotide polymorphism (SNP) calling with high confidence. WGS analysis of the specimens from episode 2 provided good coverage (99% of the genome with >30× coverage depth and 790,545 mapped reads). We determined 16 SNPs using the Wuhan-1 sequence as reference (seven of which were missense variants) (Fig. 2) and assigned the lineage of the strain (20A).

We extended the genomic analysis beyond Case A and included (i) the strain from the potential source of Case A’s second episode, her uncle, and (ii) the strains from two of her uncle’s friends (uncle’s exposure context). Moreover, we included the strain identified in Case A’s husband (potentially infected by Case A). No samples for sequencing were available from Case A’s daughter, her son-in-law, or her granddaughter. The four studied specimens yielded sequences of enough quality (>93 to 99% of the genome with >30× coverage depth and 152,354 to 525,291 mapped reads for three of the specimens and 77.53% and 290,438 for the remaining one) to allow comparisons throughout the whole genome. Case A and her husband had identical sequences and differed from those of case A’s uncle and his friends in 1 SNP (Fig. 2). This indicates that the strain involved in Case A’s reinfection was circulating in the epidemiological context of Case A’s uncle. The acquisition of one SNP, the presence of this SNP in Case A and her husband, the chronology of the cases, and the fact that Case A’s husband did not attend the religious center or meet Case A’s uncle or his friend suggest that the transmission occurred from the uncle to Case A and then from Case A to her husband.

**FIG 3:**
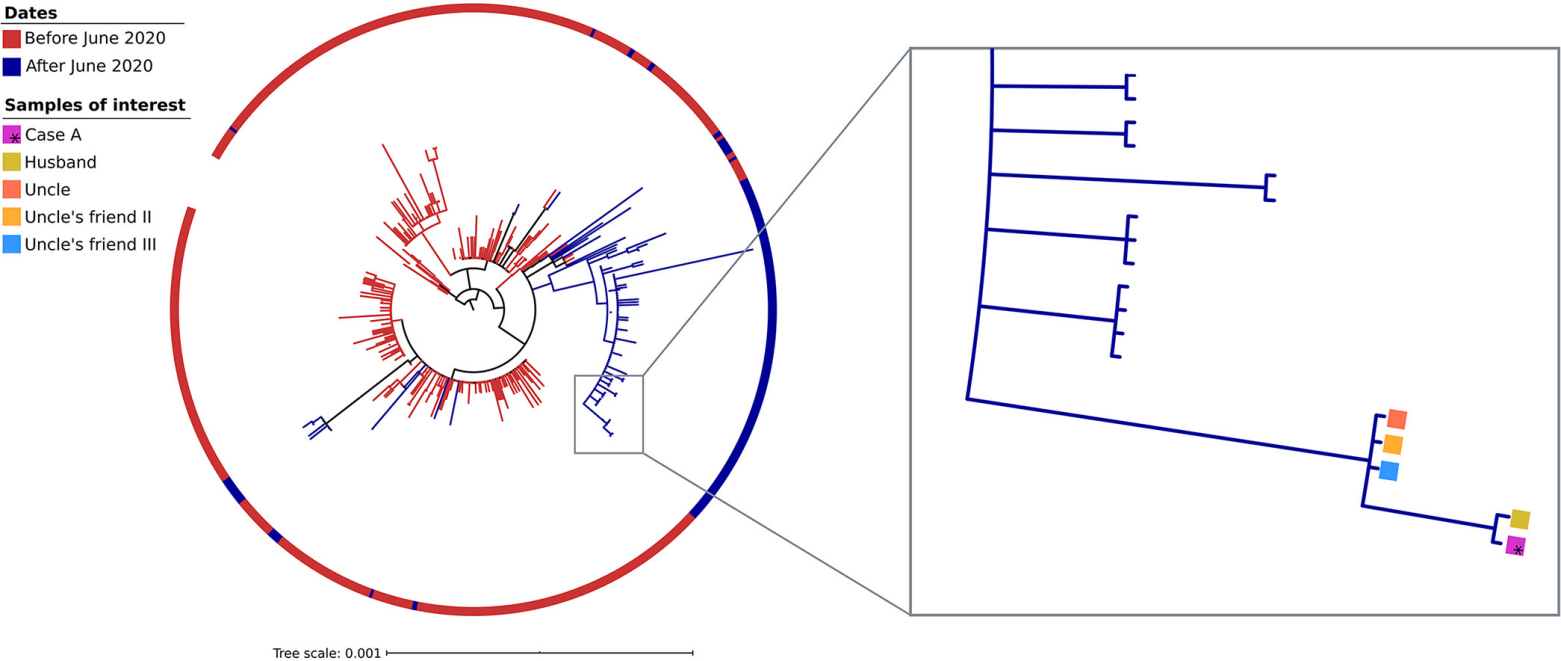
Maximum likelihood phylogenetic tree that includes the 348 sequences from samples collected from the Madrid population in the study and uploaded to GISAID on 8 October 2020. The color code in the ring indicates the sequences corresponding to the first or second COVID-19 waves (before or after June 2020). The five clustered strains, corresponding to the cases in the study (Case A, husband, uncle, and the two friends of the uncle), are highlighted with colored squares.

An extended phylogenetic analysis that included 348 strains sequenced in our institution from the same population in Madrid showed that this strain was circulating by the time Case A experienced her second COVID-19 episode in August-September 2020. The strain was part of a clade that included strains all identified after June 2020 (blue clade), and the five cases in this study shared a single proper branch within this clade (Fig. 3). The strain or related strains were not found among the strains circulating in the same population in Madrid during Case A’s first episode (end of March/beginning of April; red clades [Fig. 3]). Similarly, a dating analysis available at Nextstrain.org (https://nextstrain.org/) shows that the strain identified in the second episode belongs to a clade, 20A.EU1, that had not been described in GISAID before the end of June 2020 (Fig. S2).

10.1128/mSphere.00596-21.2 FIG S2 Dated phylogeny of the strains closely related to that identified in Case A’s second episode. The strain identified in Case A is marked with a black arrow and belongs to a phylogenetic clade, 20A.EU1, which was not circulating before the end of June 2020, ruling out its presence in the first wave. Download FIG S2, PDF file, 0.1 MB. Copyright © 2021 Pérez Lago et al. 2021 Pérez Lago et al. https://creativecommons.org/licenses/by/4.0/ This content is distributed under the terms of the Creative Commons Attribution 4.0 International license.

## DISCUSSION

Reinfection as the most likely explanation for Case A’s second episode is based on the following: (i) 4-1/2 months between her first and second episodes and confirmation by STR analysis that the specimens from the two RT-PCR-positive SARS-CoV-2 episodes came from the same patient; (ii) negative PCR after the resolution of the first episode; (iii) COVID-19 antibody seroconversion in Case A in the second episode; (iv) close physical contact without protective measures with a family member (uncle) who began with symptoms 2 days after their encounter, had a positive RT-PCR 5 days later, and started with symptoms 5 days before the onset of Case A’s symptoms in her second episode; (v) identification by WGS that the SARS-CoV-2 strains from Case A and her uncle were almost identical (1 SNP); and (vi) determination that the strain was circulating in her uncle’s epidemiological close context and in Madrid during the second episode but not at the time of the first episode.

There was no evidence of immunosuppression in Case A. She received only regular treatment for her asthma with no systemic corticosteroids. A reinfection case resembling the one in this study was reported in Belgium: female, immunocompetent, similar age (51 years), and asthma as the only risk factor, for which she was given oral corticosteroids (3).

Time lapses between the first and second episodes in previously reported COVID-19 reinfection cases (1–5, 12–15) vary considerably, from 19 days (15) (the shortest, which may also be interpreted as superinfection) to 142 (1) (the longest). For Case A, there is an interval of 140 days between the first and second episode, one of the longest reported.

To date, there is no defined pattern of severity for second episodes following reinfection. Some reinfection cases have been reported to be asymptomatic (1, 12), while others presented with milder (3) or more severe (2, 4, 5) symptoms. In the case described in this study, the second episode was much more severe. The patient developed pneumonia and had to be hospitalized.

In most reported reinfection cases, SARS-CoV-2 IgG serology is not well documented for the first episodes because during that time (March-April 2020) there was limited availability of tests. This implies that in some reinfections with seroconversion in the second episode, as in our case, it is not possible to determine whether the case did not mount an immune response in the first episode or whether antibodies were lost before the second episode was diagnosed. Similar uncertainties have been described for SARS-CoV-2 reinfections elsewhere (1, 2).

Genomics is the ultimate support to document reinfections. The most obvious way to determine a reinfection is to compare the strains from each episode. Unfortunately, and similarly to other COVID-19 reinfection reports (4), we could not obtain enough sequence information from the sample analyzed in the first episode to conclusively show that it was different from the one in the second episode.

The European Centre for Disease Prevention and Control has accepted that reinfections may be documented either by identifying genomic differences between the two episodes or by confirming that the strain from the second episode clusters with strains from the location of exposure (11). Thus, we further confirmed by genomic analysis and epidemiological enquiry that reinfection of Case A occurred due to close contact with her uncle. To the best of our knowledge, this is the first study that identifies the strain involved in a reinfection (Case A in this study) among subjects in the epidemiological close context. We not only identified the strain responsible for the reinfection of Case A (isolated from her uncle) but proved its circulation throughout her uncle’s epidemiological context, infecting three other subjects preceding the reinfection of Case A. Furthermore, we performed an extended phylogenetic analysis and determined that the strain was part of a clade of strains circulating in the same population in Madrid during the period when Case A suffered the second episode, but not during her first episode. In fact, this clade had no representative sample, at a global collection, before June 2020, and it corresponded to the clade 20A-EU1, which after emerging in Spain at the end of June successfully spread throughout Europe (16). The reinfection event, involving five cases, occupies an independent branch within the clade.

Understanding Case A’s epidemiological context allows us to show for the first time the onward transmission leading to a reinfection and to report subsequent transmission from a reinfection case. The identity of Case A and her husband’s strains, including a private marker SNP not shared by other cases, the chronology of symptom onset, and the confirmation that Case A’s husband did not interact with her uncle or his friends and did not attend the religious center confirmed Case A as the sole source for the infection of her husband.

There was a likely noteworthy transmission from Case A after her reinfection, beyond her husband, deduced from the sequential chronology of symptoms and positive RT-PCRs obtained for her daughter, son-in-law, and four grandchildren. Unfortunately, no samples were available from these subjects as they were diagnosed in another institution where specimens were not stored. Therefore, the lack of sequencing data from the son-in-law and grandchildren impeded us from fully supporting their involvement in the transmission chain.

Here, we describe the first complete analysis of the epidemiological scenario around a reinfection by SARS-CoV-2 supported by host genetic analysis, viral genomic analysis, phylogenomic population analysis, and in-depth epidemiological investigation. This extensive approach documents a much more severe second COVID-19 episode in a woman without risk factors, except for asthma, after exposure through a close contact to a SARS-CoV-2 strain actively transmitted in the same setting/population. Once reinfected, our case was responsible for an extensive transmission among family members.

## MATERIALS AND METHODS

The study was approved by the ethical research committee of Gregorio Marañón Hospital (REF: MICRO.HGUGM.2020-042). Informed consent was obtained from the patient for publication of this case report.

### Specimens.

The remnants from diagnostic nasopharyngeal SARS-CoV-2 RT-PCR-positive swabs were aliquoted and stored at −70°C until analysis.

### Diagnostic RT-PCR.

RNA was extracted and purified from 300 μl of nasopharyngeal exudates using the EasyMag (BioMérieux, France) (specimen from April) or KingFisher (Thermo Fisher Scientific, Waltham, MA) (specimens from August-September) instruments.

Next, real-time RT-PCRs were performed. The novel coronavirus (2019-nCoV) nucleic acid diagnostic kit (Sansure Biotech, China) was used for the April specimen, and the TaqPath COVID-19 CE-IVD RT-PCR kit (ThermoFisher Scientific, USA) was used for the August-September specimens.

### Whole-genome sequencing.

Eleven microliters of RNA was used as the template for reverse transcription with Invitrogen SuperScript IV reverse transcriptase (ThermoFisher Scientific, MA, USA) and random hexamers (ThermoFisher Scientific, MA, USA). Whole-genome amplification of the coronavirus was done with the Artic_nCov-2019_V3 panel of primers (Integrated DNA Technologies, Inc., Coralville, IA, USA) (artic.network/ncov-2019) and the Q5 Hot Start DNA polymerase enzyme (New England Biolabs, Ipswich, MA, USA). Libraries were prepared using the Nextera Flex DNA library preparation kit (Illumina Inc., CA, USA), following the manufacturer’s instructions.

Libraries were quantified with the Quantus fluorometer (Promega, WI, USA) before being pooled at an equimolar concentration (4 nM). Next, libraries were sequenced in pools of up to 17 libraries on the MiSeq system (Illumina Inc., CA, USA) using the MiSeq Reagent Micro kit v2 (2 × 151 bp) or in pools of up to 96 libraries with the MiSeq reagent (2 × 201 bp).

A bioinformatics pipeline developed by the SeqCOVID consortium was applied to analyze the sequencing reads. The pipeline is based on the computational tool iVar (17) which can be accessed at https://gitlab.com/fisabio-ngs/sars-cov2-mapping. Briefly, the pipeline goes through the following steps: (i) removal of human reads with Kraken (18), (ii) preprocessing of the fastq files using fastp (19) v 0.20.1 (arguments: –cut tail, –cut-window-size, –cut-mean-quality, -max_len1, -max_len2), (iii) mapping and variant calling using iVar version 1.2, and (iv) quality control assessment with MultiQC (20).

The consensus sequences generated by this pipeline were aligned against the SARS-CoV-2 reference sequence (21) with MAFFT (22). Problematic positions were masked using the mask_alignment.py script from the repository maintained by Rob Lanfear (https://zenodo.org/record/4069557#.X37nuXUzaWg). Clade nomenclature was assigned using Nextstrain (16).

A maximum-likelihood phylogenetic tree was reconstructed. We included all masked positions from the sequences in the study with those from the same population in Madrid uploaded to GISAID until 8 October 2020. The analysis was restricted to the case’s health district. We used IQ-tree v2 (23), with the GTR as the substitution model and the czb option, and rooted the tree with the Wuhan-1 reference sequence.

### STR analysis.

Short tandem repeat (STR)-PCR (Mentype Chimera; Biotype, Germany) was applied for the human identity testing analysis. For this, the specimens used for SARS-CoV-2 genome sequencing were employed. We examined 12 noncoding STR loci and the gender-specific locus amelogenin (see Table S1 in the supplemental material), labeled with three different dyes (6-carboxyfluorescein [FAM], BTG, or BTY). The selected loci have a very high rate of heterozygosity and balanced allelic distribution (24). PCR was performed with 0.2 to 1 ng of genomic DNA with the Mentype Chimera PCR amplification kit (Biotype, Germany), the GeneAmp PCR System 9700 Thermal Cycler (Applied Biosystems), and subsequent capillary electrophoresis in a Genetic Analyzer 3130*xl* (Applied Biosystems) as recommended by the manufacturer.

10.1128/mSphere.00596-21.3 TABLE S1 Noncoding STR loci and the gender-specific locus amelogenin used for the short tandem repeat analysis. Download Table S1, DOCX file, 0.02 MB. Copyright © 2021 Pérez Lago et al. 2021 Pérez Lago et al. https://creativecommons.org/licenses/by/4.0/ This content is distributed under the terms of the Creative Commons Attribution 4.0 International license.

### Data availability.

The data that support the findings of this study (Fastq files) are publicly available. Fastq files above the GISAID thresholds were deposited at GISAID (hCoV-19/Spain/MD-IBV-99007733/2020, hCoV-19/Spain/MD-IBV-99007151/2020, hCoV-19/Spain/MD-IBV-99007734/2020, and hCoV-19/Spain/MD-IBV-99007170/2020). All sequences were also deposited at the ENA (European Nucleotide Archive; https://www.ebi.ac.uk/ena/browser/home) (ERR5698024, ERR5697187, ERR6459974, ERR5698025, and ERR5697254).
