# Supplementary figures and images for: Complete Analysis of the Epidemiological Scenario around a SARS-CoV-2 Reinfection: Previous Infection Events and Subsequent Transmission
Source: mSphere. 2021 Sep 8;6(5):e00596-21. doi: 10.1128/mSphere.00596-21 (PMC8550076; doi:10.1128/mSphere.00596-21)

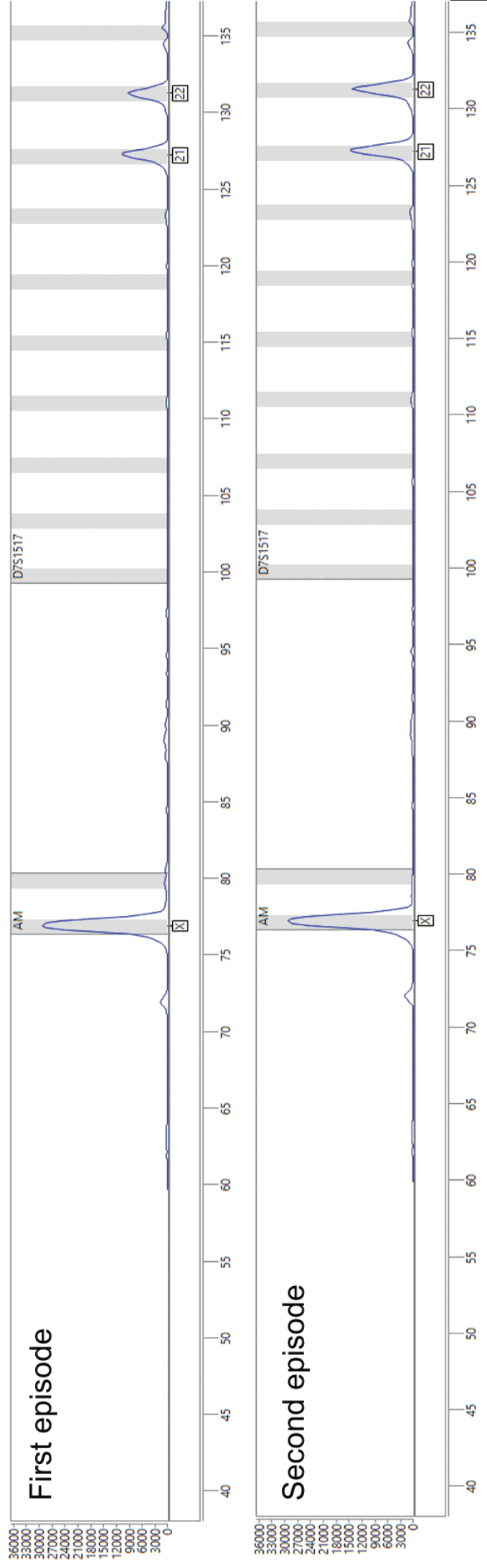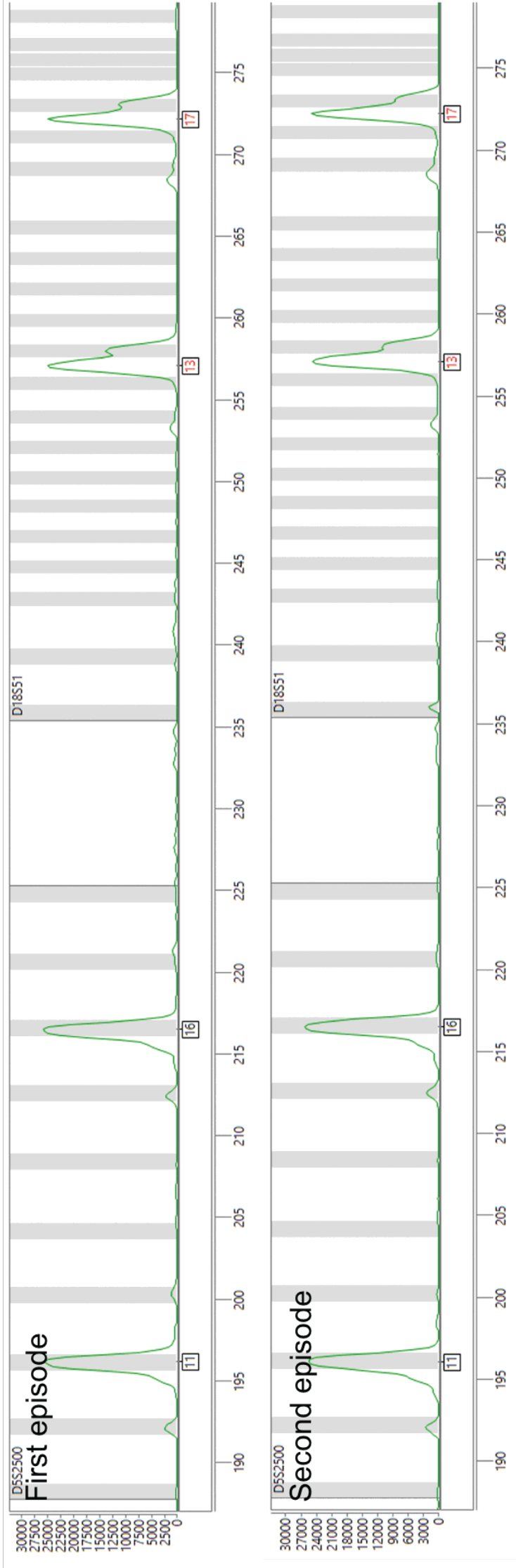

Supplement: FIG S1 [file msphere.00596-21-sf001.pdf]

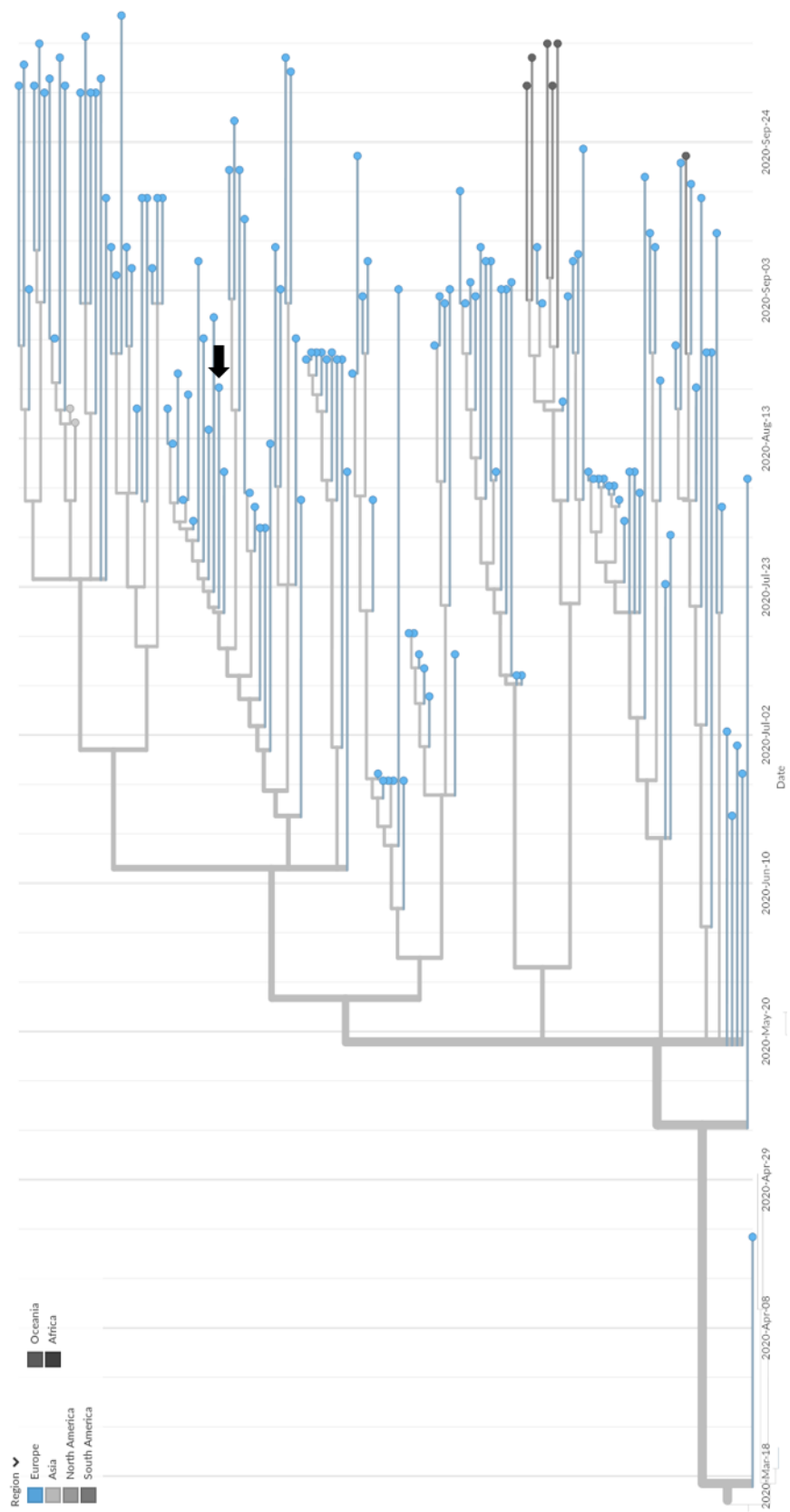

Supplement: FIG S2 [file msphere.00596-21-sf002.pdf]
