# Supplementary material for: Complete Analysis of the Epidemiological Scenario around a SARS-CoV-2 Reinfection: Previous Infection Events and Subsequent Transmission
Source: mSphere. 2021 Sep 8;6(5):e00596-21. doi: 10.1128/mSphere.00596-21 (PMC8550076; doi:10.1128/mSphere.00596-21)
Supplement: TABLE S1 [file msphere.00596-21-st001.docx]

**Supplementary Table S1**

| **Locus** | **GenBank**  **accession** | **Repeated motif**  **of the reference allele** | **Reference**  **allele** | **Allele range** | **Chromosomal Mapping** |
| --- | --- | --- | --- | --- | --- |
| D2S1360 | G08130 | [TATC]9 [TGTC]9 [TATC]5 | 23 | 19-32 | 2p24-p22 |
| D3S1744 | G08246 | [TCTA]2 TA[TCTA]12 TCA [TCTA]2 | 16 | 13-22 | 3p24 |
| D4S2366 | G08339 | [ATAG]9 ATTG [ATAG]2 | 12 | 9-15 | 4p16-15.2 |
| D5S2500 | G08468 | [ATAG]12 | 12 | 9-18 | 5q11.2 |
| D6S474 | G08540 | [TAGA]5 TGA [TAGA]12 | 17 | 11-20 | 6q21-22 |
| D7S1517 | G18365 | [GAAA]11 CAAA [GAAA]2 CAAA [GAAA]2 | 17 | 14-31 | 7q31.33 |
| D8S1132 | G08685 | [TCTA]9 TCA [TCTA]9 TCTGTCTA | 20 | 12.1-27 | 8q23.1 |
| D10S2325 | G08790 | [TCTTA]12 | 12 | 6-23 | 10p12 |
| D12S391 | G08921 | [AGAT]5 GAT [AGAT]7 [AGAC]6 AGAT | 19.3 | 13-28 | 12p13.2 |
| D18S51 | L18333 | [AGAA]13 | 13 | 5.3-42 | 18q21.3 |
| D21S2055 | G27274 | [CTAT]2 CTAA [CTAT]9 CTA [CTAT]3 TAT  [CTAT]3 TAT [CTAT]4 CAT[CTAT]2 | 24 | 16.1-39 | 21q22 |
| SE33 (ACTBP2) | NG000840 | [AAAG]9 AA [AAAG]16 | 25.2 | 3-50 | 6q14.2 |
| Amelogenin X | M55418 |  | | | Xp22.1-22.3 |
| Amelogenin Y | M55419 |  | | | Yp11.2 |
